# lociNGS: A Lightweight Alternative for Assessing Suitability of Next-Generation Loci for Evolutionary Analysis

**DOI:** 10.1371/journal.pone.0046847

**Published:** 2012-10-10

**Authors:** Sarah M. Hird

**Affiliations:** 1 Department of Biological Sciences, Louisiana State University, Baton Rouge, Louisiana, United States of America; 2 Museum of Natural Science, Louisiana State University, Baton Rouge, Louisiana, United States of America; University of California Riverside, United States of America

## Abstract

Genomic enrichment methods and next-generation sequencing produce uneven coverage for the portions of the genome (the loci) they target; this information is essential for ascertaining the suitability of each locus for further analysis. lociNGS is a user-friendly accessory program that takes multi-FASTA formatted loci, next-generation sequence alignments and demographic data as input and collates, displays and outputs information about the data. Summary information includes the parameters coverage per locus, coverage per individual and number of polymorphic sites, among others. The program can output the raw sequences used to call loci from next-generation sequencing data. lociNGS also reformats subsets of loci in three commonly used formats for multi-locus phylogeographic and population genetics analyses – NEXUS, IMa2 and Migrate. lociNGS is available at https://github.com/SHird/lociNGS and is dependent on installation of MongoDB (freely available at http://www.mongodb.org/downloads). lociNGS is written in Python and is supported on MacOSX and Unix; it is distributed under a GNU General Public License.

## Introduction

To apply the immense sequencing capabilities of next-generation sequencing (NGS) technologies to population-level questions (i.e., those that require multi-locus, multi-individual data), genome enrichment methods are frequently employed. These methods aim to sample the genome at a reproducible subset of markers that can be obtained from many individuals and reduced to genotype (i.e., a set of phased alleles). Examples of these methods include amplicon sequencing [1], RAD-tags [2], complexity reduction of multilocus sequences (or CRoPS) [3] and sequence capture [4]; for a review of NGS methods suitable for multi-locus studies, see [5]. Genome enrichment methods often utilize a known or constructed reference for easing alignment of sequencing reads. Genotypes can then be called from the alignments, using a variety of bioinformatics methods (e.g., [6], [7]). This results in next-generation alignments to a reference and a set of loci for the individuals in the study; the loci can then be used in standard phylogeographic, phylogenetic or population genetic studies or other multi-locus analyses (e.g., [8];[9]). Prior to analysis, however, researchers must determine which loci are suitable for the questions being asked by assessing key parameters such as coverage and number of polymorphic sites or whether all populations are represented.

Current NGS file types are efficient at manipulating and storing alignment data but the parameters of interest are difficult to extract and can require custom bioinformatics scripts. Additionally, these file types are not useable in downstream analyses. Although large-scale, comprehensive programs like the Genome Analysis Toolkit (GATK) [10] can calculate coverage, if the parameters of interest are limited and include coverage per locus and coverage per individual, these programs are more heavy-duty and time-intensive than a user may want to invest. lociNGS is a lightweight, easy to use program that displays and outputs key parameters for researchers interested in multi-locus analysis of genotypes.

As more NGS papers come out, it should be standard to report summary statistics about coverage and polymorphism, in addition to the already standard number of total and high quality reads. Furthermore, as sequencing capacity continues to increase, the number of loci and number of individuals in a dataset will as well. Easily accessing, summarizing and reporting these parameters are important steps toward streamlining analysis and understanding large multi-locus datasets. lociNGS does not analyze any of the user-supplied data – it simply reports and exports summarized information about the dataset contained in the input files that is difficult to extract manually.

## Methods

### Overview

**Figure 1:**
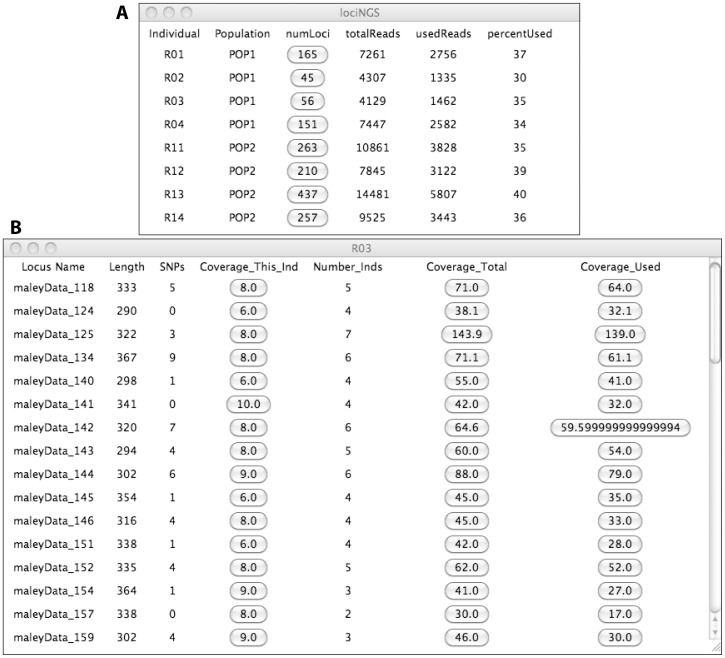
Screen shots of lociNGS. Data include 8 individuals (rails); summarized data for the whole dataset shown in the summary screen (**A**) and one example of an individual (R03) screen shows parameters associated with individuals (**B**). Details of the column headings are in Table 1.

lociNGS was designed for use with multi-locus, multi-individual datasets generated through NGS. It collates information about loci, alignments and demographic data so that users can view summarized information about the genetic data (Table 1; Fig. 1) on the same screen as taxonomic and field data (e.g., subspecies, sampling locality, gender, etc.). In this way, one may assess the suitability of the data for further analysis.

**Table 1:** lociNGS parameters for the summary screen (Sum; Fig. 1a) and the individual screen (Ind; Fig. 1b).

Screen^a^	Parameter^b^	From^c^	*^d^	Definition
Sum	Individual	Demo		The individual's name
Sum	Population	Demo		The individual's population of origin
Sum	numLoci	Align	*	The number of loci called for each individual
Sum	totalReads	Align		Total number of reads sequenced in each individual
Sum	usedReads	Align		Total number of reads used for calling loci in this individual
Sum	percentUsed	lociNGS		UsedReads/TotalReads
Ind	LocusName	Loci		The name of the locus
Ind	Length	Loci		Number of bases in the locus
Ind	SNPs	Loci		Number of polymorphic sites
Ind	Number_Inds	Loci		Number of individuals called for this locus
Ind	Coverage_This_Ind	Align	**	Coverage for this locus in this individual
Ind	Coverage_Total	Align	**	Total coverage across individuals for this locus
Ind	Coverage_Used	Align	**	Total coverage for all individuals used in final locus

a Which screen the data are displayed on, the summary or the locus screen.

b Column header displayed in program; see Figure 1.

c Which input file the data are derived from, demographic data (demo), SAM/BAM alignments (Align), multi-FASTA locus files (loci) or calculated by lociNGS.

d * indicates this column's data serve as a button to pull up locus screen; **indicates this column's data serves as a button to print the corresponding reads to a multi-FASTA file.

The program has two types of display screens, both in table format. The “summary screen” contains demographic data, number of loci per individual (numLoci), total number of reads sequenced, number of reads used (along with the percentage of total). The numLoci data serve as buttons that open the corresponding “individual screen”. This screen displays specific information about all the loci found in an individual, including length of the locus, number of polymorphic sites, number of individuals sequenced for that locus and coverage (for the individual, for all individuals, and for only the individuals with high enough coverage to be called). Each of the coverage categories serves as buttons that print the corresponding raw data in multi-FASTA format.

### Program Input

**Figure 2:**
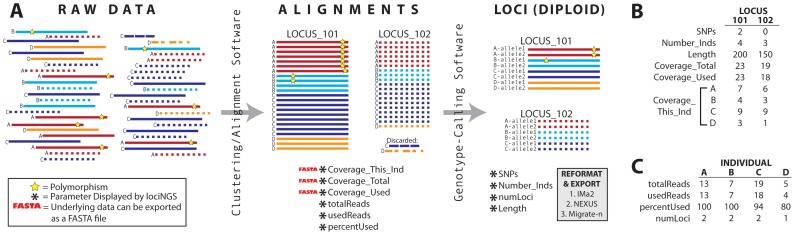
How the data are generated, where the parameters come from and example data. (**A**) Letters represent individuals and lines represent sequences; there are four individuals and two loci. Raw data from the sequencer is put through an alignment or clustering program to collect reads into alignments. From each alignment file, lociNGS reports totalReads, usedReads, percent reads used (percentUsed), Coverage_This_Ind, Coverage_Total and Coverage_Used; lociNGS will also export the data underlying the coverage parameters in FASTA format. Genotype-calling software will reduce sequence reads to loci (phased alleles). lociNGS uses these loci to report SNPs, Number_Inds, numLoci and Length; the program can reformat the loci into IMa2, NEXUS or Migrate formats. For further explanation of the parameters, see Table 1. (**B**) The parameter values for the two loci (LOCUS_101 and LOCUS_102) in this example. (**C**) The parameter values for the four individuals (A,B,C,D) in this example.

lociNGS takes three categories of input: NGS alignment files, locus files (Fig. 2) and a demographic data file. When using genomic enrichment methods (or genome assembly methods), an alignment of the raw sequencing reads to a reference genome is often constructed using clustering or alignment programs (e.g., Geneious [11], Galaxy [12], Velvet [13], etc.). One common format for these alignments is SAM (Sequence Alignment/Map [14]) format or its binary version, BAM. These alignments contain a lot of information about the sequences and are lociNGS's source for many of the coverage and sequence data parameters (see Table 1). For input to the program, the alignment files need to be in sorted, indexed BAM format; the program samtools [14] can be used to convert SAM to BAM, sort and index the reads, if necessary.

Many traditional evolutionary analyses require individual loci that contain phased, homologous alleles for the individuals in the dataset. To get from alignments to loci, genotype-calling software is required (e.g. PRGmatic [6], STACKS [7], GATK [10], [15], etc.). The loci are analogous to traditional Sanger sequencing loci and should be in multi-FASTA format. The locus files are the source for the SNP parameter as well as the locus names and length (see Table 1).

Finally, a demographic text file is required that, at a minimum, assigns each individual to a population; designating populations is frequently important in population level questions and is required because the output formats are capable of outputting a subset of populations or individuals. However, if this information is unknown or the user does not need the IMa2 or migrate output options, population can be set to something meaningless and the program will function properly.

### Program Output

lociNGS outputs several different types of data. First, a table of all the information displayed to the user may be printed as a tab-delimited text file. This can then be edited with a spreadsheet or text-editing program to calculate averages, construct graphics, sort the data, etc.

Second, the raw sequences that were used to call a locus may be exported for an individual, for all individuals or just the individuals that were used in the final dataset; this information is contained in the alignment files but difficult to extract manually. These data are FASTA formatted.

Third, users may reformat a subset of populations or individuals into NEXUS [16], IMa2 [17] or Migrate [18] formats. These three formats are highly specific and are used in population genetics programs that can analyze large, multi-locus datasets. In addition, these formats can be rather time consuming to produce by hand or require custom scripts to produce for more than a few loci. lociNGS automates and combines the selection of loci and the construction of the appropriate input files. Under the export menu of the program, users select either populations or individuals they would like to include in the output of these formats; lociNGS then searches all the loci that contain at least one individual from the populations selected or all individuals selected.

The location of all exported files is logged to the screen and each has a unique file name.

### Test Data

There is a small test dataset provided with the lociNGS distribution. This dataset includes four individuals at five loci. A copy of the exact parameter values displayed by lociNGS with the test data is included as supplemental material (Table S1).

### Program Implementation

lociNGS is written in Python for a Unix-based system (e.g., MacOSX). It requires MongoDB as a separately installed program. lociNGS uses the Tkinter class of Python for a user-friendly graphical user interface. A modified version of seqlite (available: http://www.mbari.org/staff/haddock/scripts/) calls polymorphic sites from the aligned locus files; this tool works by simply counting the variable sites in an aligned FASTA file. The BAM files are not considered in the number of SNPs. The User Manual is included as a Supplementary File (Document S1).

## An Example: Using lociNGS in Phylogeography

**Figure 3:**
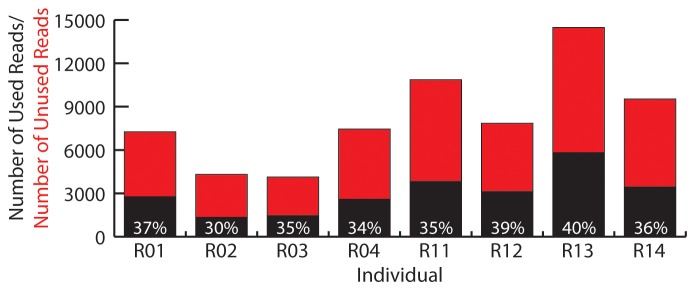
Number of reads per individual. Black portion of bars represents reads aligned to the reference; red portion accounts for unused reads. Percentage of reads used is shown in white text.

For many evolutionary analyses, a phased set of alleles is required as input; many NGS molecular and computational methods are now capable of producing such datasets. For example, McCormack et al. [8] generated restriction-digested fragments sequenced on a Roche 454 platform for two species of rails (*Rallus longirostris* and *R. elegans*) to identify fixed genetic differences in a bird hybrid zone; in this section I walk through a subset of their dataset that contains 4 individuals from each species (*R. longirostris* = R01, R02, R03, R04; *R. elegans* = R11, R12, R13, R14). The data was quality controlled and analyzed with PRGmatic [6], then loaded in to lociNGS. The summary screen (Fig. 1a), which can be exported as a tab-delimited text file, informs the user of how efficient the method was, in terms of how many reads were aligned to the reference genome compared to total number of reads (Fig. 3). It also displays the total number of loci that each individual belongs to; these data functions as a button that opens the individual screen for the given individual (Fig. 1b).

**Figure 4:**
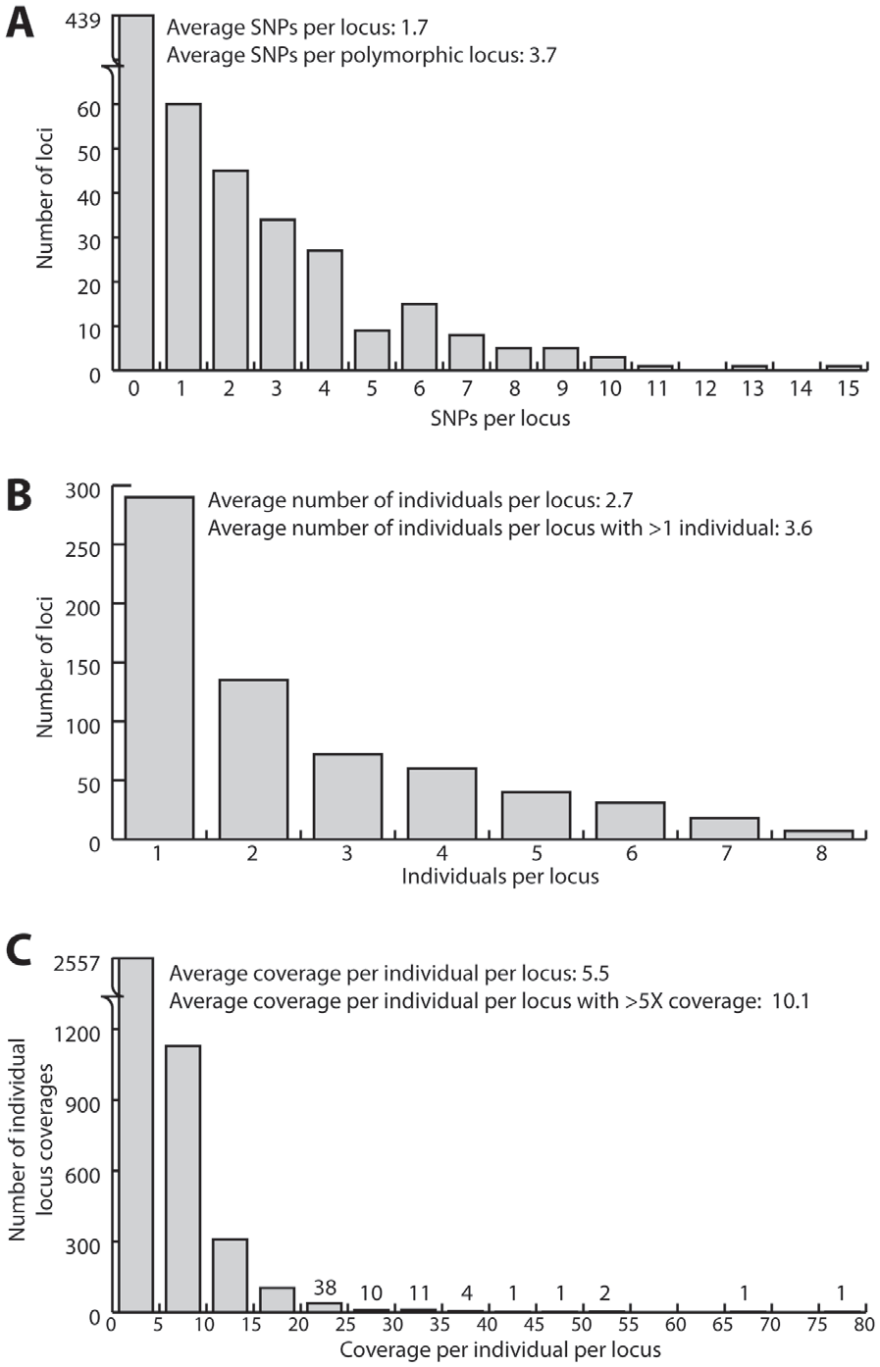
Summary histograms of important parameters in the rail dataset. Number of polymorphic sites (**A**), individuals present in each locus (**B**), individual coverage on a per locus basis (**C**). Note the scale of the dependent axis changes on (**A**) and (**C**).

The individual screen contains detailed information about each of the loci with links to the raw data that make up each locus (Fig. 1b). Exporting this data as a tab-delimited text file allows the user to determine the distributions of polymorphic sites (Fig. 4a), number of individuals (Fig. 4b) and coverage per individual (Fig. 4c) across all loci. One can also assess how well each individual performed, by calculating average coverage. One may use this information to decide which individuals are worth resequencing with custom primers (to fill in their data matrix) or how to prune their dataset to the most complete or informative loci.

**Figure 5:**
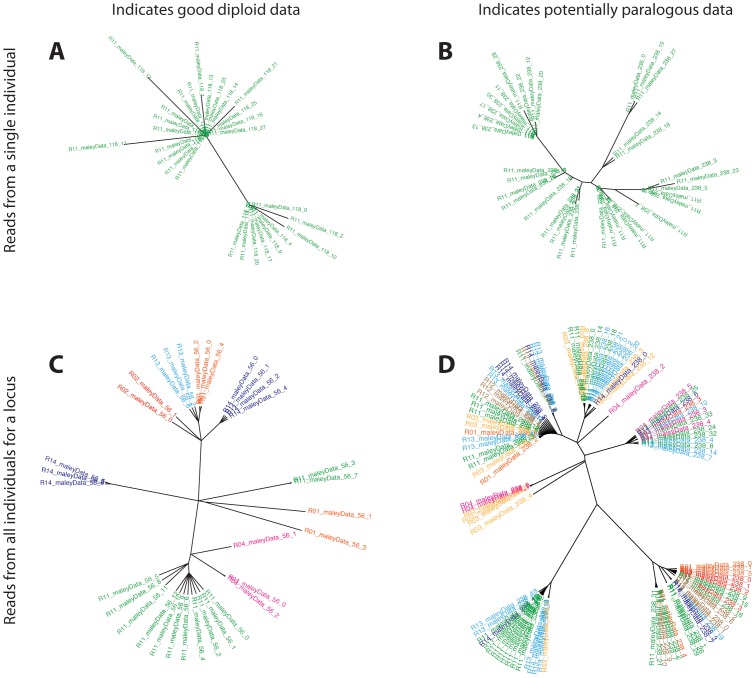
Neighbor-joining trees of aligned reads (reads output from the program) to help assess copy number. Shown are reads from one individual (**A**, **B**) and all the reads for a locus (**C**, **D**). Both (**A**) and (**C**) imply single copy loci; in (**A**) there are only two major clades and in (**C**) the reads for each individual, as shown by the different colors, belong to two clades at the most. Both (**B**) and (**D**) indicate potential multi-copy loci; in (B), there are greater than two clades and in (**D**) the reads for each individual, as shown by the different colors, are frequently distributed across greater than two clades.

If a particular locus has more polymorphic sites than one might expect by the processes of natural selection or drift, the user can output the sequence reads that compose the raw data to investigate underlying copy number. With the raw read data, an alignment and phylogenetic tree can be estimated from either a single locus for one individual or all the reads underlying a single locus from all individuals (Fig. 5), but analysis of the raw reads is up to the user. For these data, I used Muscle [19] for alignment (using all defaults) and Geneious [11] to construct a neighbor-joining tree (using an HKY model of genetic distance and no outgroup). An analysis like this is very quick and although more sophisticated phylogenetic algorithms exist, for the purposes of assessing number of clades, these methods worked well. Once a tree has been constructed, if there are two (or fewer) major clades for each individual, it is likely that the sequences derive from a single diploid locus (Fig. 5a). However, if there are more clades than the ploidy of the organism allows, there may be multiple genomic sources of the data (Fig. 5b). One can also assess paralogy in the reads from all individuals at a locus: if all the reads from each individual belong to two or fewer clades, the locus is likely single copy (Fig. 5c). However, if one or more individuals belong to multiple clades, the underlying copy number may not be one (Fig. 5d).

Finally, lociNGS will export the data in three formats for input to evolutionary analysis programs. Users select exportation of either individuals or populations. The program searches for all loci that contain at least one individual from each of the selected categories. In other words – if all individuals are selected, only the loci that contain all individuals will be reformatted and printed. If all populations are selected, only the loci that contain at least one individual from each population will be reformatted and printed.

Altogether, these simple functions provide the user with an overall sense of how their method and data perform at a basic level.

## Conclusions

With the ever-increasing amount of data that is gathered with NGS, it is important to assess the suitability of the reads for further analysis. lociNGS provides a simple and quick way to determine which loci and which individuals have enough coverage and polymorphism to use in evolutionary analysis. Furthermore, the program automatically converts suitable loci to several file formats that are common in evolutionary analysis and time consuming when done by hand. Small, easy to use programs designed for a specific task allow researchers to customize their workflow and minimize or eliminate the learning curve for complex programs.

## Supporting Information

Table S1 **Expected results from test data included with lociNGS.** The exact results that the program should output if the test data is input into the program.
(PDF)

Document S1 **README file for lociNGS.** The README file contains detailed information about installation, input options, output options and troubleshooting.
(TXT)
