# Supplementary material for: lociNGS: A Lightweight Alternative for Assessing Suitability of Next-Generation Loci for Evolutionary Analysis
Source: PLoS One. 2012 Oct 10;7(10):e46847. doi: 10.1371/journal.pone.0046847 (PMC3468592; doi:10.1371/journal.pone.0046847)
Supplement: Table S1 — Expected results from test data included with lociNGS. The exact results that the program should output if the test data is input into the program. (PDF) [file pone.0046847.s001.pdf]

Table S1: Expected Results From Test Data Included With lociNGS
*These are fake data with no biological meaning, but confirm that the program is properly installed.The screens should look like Figure 1A and 1B from the main text (Hird 2012).

**Summary screen**	Individual	Population	numLoci	totalReads	usedReads	percentUsed	Longitude	Latitude	Location	Species
	testA	POP1	5	3472	1272	36	-109.876	45.678	NoPlace, TX	Tamias bunkus
	testB	POP2	4	1753	659	37	-109.876	45.678	NoPlace, TX	Tamias bunkus
	testC	POP1	5	5138	1881	36	-109.876	45.678	NoPlace, TX	Tamias bunkus
	testD	POP3	5	2139	593	27	-109.876	45.678	NoPlace, TX	Tamias tamias

Individual screens

testA	Locus Name	Length	SNPs	Coverage_This_Ind	Number_Inds	Coverage_Total	Coverage_Used
	false_1	136	1	170.0	3	630.0	630.0
	false_2	145	0	31.0	4	81.0	74.0
	false_3	282	4	210.0	4	720.0	720.0
	false_4	191	1	250.0	4	761.0	761.0
	false_5	177	7	180.0	4	260.0	260.0

testB	Locus Name	Length	SNPs	Coverage_This_Ind	Number_Inds	Coverage_Total	Coverage_Used
	false_1	136	1	170.0	3	630.0	630.0
	false_3	282	4	210.0	4	720.0	720.0
	false_4	191	1	250.0	4	761.0	761.0
	false_5	177	7	180.0	4	260.0	260.0

testC	Locus Name	Length	SNPs	Coverage_This_Ind	Number_Inds	Coverage_Total	Coverage_Used
	false_1	136	1	170.0	3	630.0	630.0
	false_2	145	0	31.0	4	81.0	74.0
	false_3	282	4	210.0	4	720.0	720.0
	false_4	191	1	250.0	4	761.0	761.0
	false_5	177	7	180.0	4	260.0	260.0

testD	Locus Name	Length	SNPs	Coverage_This_Ind	Number_Inds	Coverage_Total	Coverage_Used
	false_1	136	1	170.0	3	630.0	630.0
	false_2	145	0	31.0	4	81.0	74.0
	false_3	282	4	210.0	4	720.0	720.0
	false_4	191	1	250.0	4	761.0	761.0
	false_5	177	7	180.0	4	260.0	260.0
